# Prevalence of Low Serum Vitamin D Levels in Patients Presenting With Androgenetic Alopecia: A Review

**DOI:** 10.7759/cureus.20431

**Published:** 2021-12-15

**Authors:** Zainab Zubair, Ketan Kantamaneni, Krishi Jalla, Mahvish Renzu, Rahul Jena, Ruchi Jain, Suchitra Muralidharan, Vijaya Lakshmi Yanamala, Michael Alfonso

**Affiliations:** 1 General Surgery, California Institute of Behavioral Neurosciences & Psychology, Fairfield, USA; 2 Surgery, California Institute of Behavioral Neurosciences & Psychology, Fairfield, USA; 3 Internal Medicine, California Institute of Behavioral Neurosciences & Psychology, Fairfield, USA; 4 Medicine, California Institute of Behavioral Neurosciences & Psychology, Fairfield, USA; 5 Diagnostic Radiology, California Institute of Behavioral Neurosciences & Psychology, Fairfield, USA; 6 School of Medicine, Universidad del Rosario, Bogota, COL

**Keywords:** ergocalciferols, vitamin d, non-scarring alopecia, androgenetic alopecia, alopecia

## Abstract

The role of vitamin D receptor (VDR) has been well established and extensively studied in the hair cycle. Its deficiency is also closely linked to several types of alopecia, including alopecia areata, telogen effluvium, and androgenetic alopecia (AGA). Since there is limited research on the correlation between androgenetic alopecia and low serum vitamin D levels, our review aims to find relevant articles and comprehensively present them. A review of the literature was performed to gain insight into AGA. Specifically, PubMed and Google Scholar databases were searched to identify any relevant articles with a focus on androgenetic alopecia, male pattern baldness, and serum vitamin D levels. References within the included articles were also reviewed and taken into the study if found appropriate. All articles that met the inclusion criteria were analyzed for demographics, clinical, laboratory, radiographic, treatment, and outcomes data. We found 13 relevant studies that elucidated the relationship between low serum vitamin D levels and androgenetic alopecia and included them in the review. We concluded that serum vitamin D might be a possible parameter for diagnosing the onset and severity of AGA. Vitamin D supplementation has proven to be useful in the regrowth of hair in non-human subjects. Vitamin D could be a valid therapeutic approach, such as topical vitamin D (calcipotriol) seems to be a good treatment option to regrow hair follicles and prevent miniaturization of follicles due to androgenetic alopecia.

## Introduction and background

Alopecia refers to the loss of hair from any part of the body. There are two main types of alopecia; scarring and non-scarring. Scarring alopecia is further divided into six subtypes, including androgenetic alopecia (AGA), telogen effluvium, alopecia areata (AA), traumatic alopecia, tinea capitis, and anagen effluvium [1]. AGA is the most common type of progressive hair loss [2]. It is defined as androgen-related progressive thinning of hair in a defined pattern [3]. AGA is further divided into two types: male and female pattern hair loss. Both have different patterns but are the most common types of baldness in both genders [4]. AGA is classified according to the Norwood Hamilton scale. The Hamilton classification system describes the predominant course in men as a receding frontal hairline with bitemporal hair loss that merges with vertex thinning [5]. The age of onset is usually the third and fourth decades, but the hair loss starts at puberty [6]. Male pattern hair loss has become increasingly prevalent among younger populations. In a study, including male subjects 18-49 years of age, it was found that 42% of included subjects suffered from moderate to extensive hair loss [7]. Premature AGA can lead to deep psychological effects and self-esteem issues [8]. Hair thinning results from the testosterone metabolite (dihydrotestosterone) acting on androgen-sensitive hair follicles [9]. 

**Figure 1:**
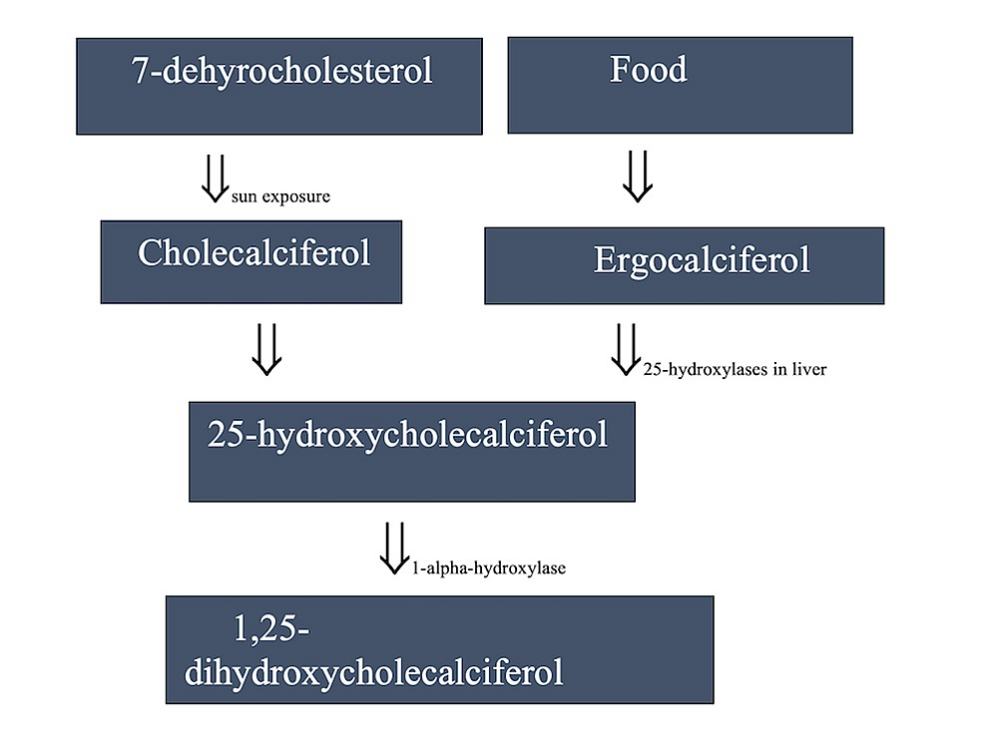
The synthesis of Vitamin D in the body

The discovery of vitamin D and the elimination of rickets as a major medical problem is considered one of the greatest achievements in medicine [10]. The two main sources of vitamin D are cutaneous production from exposure to sunlight and oral intake, including both dietary and supplementation [11]. Vitamin D3 is formed in keratinocytes from 7-dehydrocholesterol when exposed to UVB (Ultraviolet-B) radiations. The liver and other tissues metabolize vitamin D to 25 hydroxycholecalciferol (25(OH)D), which is then further metabolized to 1,25-dihydroxycholecalciferol (1,25(OH)2D) in the kidney [12]. Figure 1 demonstrates the synthesis of the active form of vitamin D in the body. 25(OH)D is considered the storage form of vitamin D, while 1,25-(OH)2D is considered the active form. Vitamin D asserts its effects through the VDR (vitamin D receptor), which is a member of the superfamily of the ligand-dependent nuclear receptor [13]. Currently, the internationally approved method of assessing vitamin D levels is measuring the levels of serum 25(OH)D [14]. Vitamin D deficiency is defined as serum 25(OH)D2 concentration less than 20-25 nmol/l, and vitamin D insufficiency is defined as a serum 25(OH)D2 concentration between 25 and 75 nmol/l [15]. 

The maintenance of hair follicles postnatally is dependent on the integrity of the dermis, the epidermis, and the normal hair cycle [16]. The hair cycle consists of four phases, anagen, catagen and telogen, and exogen. Anagen growth is the active phase in which the hair follicle takes an onion-like shape and works to produce the hair fibre. Catagen, which can last a few weeks, the hair follicle undergoes apoptosis-driven regression and loses about one-sixth of its standard diameter. Next is the telogen or resting phase of the hair cycle, in which the hair follicle is dormant, and growth of the hair shaft does not occur. This phase lasts several days (eyelash hair) to several years (scalp hair) [17]. The final stage in the hair cycle is exogen, which can last 2-5 months. In this stage, hair falls out of its follicle. After this, the hair cycle begins again.

Vitamin D is a hair follicle differentiation promoter. Correlation of vitamin D levels has been seen among patients with hereditary VDR deficiency with alopecia [18]. In a study conducted by Demay et al., VDR null mice were unable to initiate a new hair cycle after a period of morphogenesis [19]. However, there is limited evidence and a huge research gap pointing to the correlation between low serum vitamin D levels and its effect on the onset of AGA. This review aims to establish the correlation between low serum 25(OH)D and patients presenting with AGA.

## Review

Methods

A literature search was performed using the MeSH search strategy, and keywords “Androgenetic alopecia”, “Male pattern baldness”, "Serum vitamin D” were used. Studies relevant to the correlation between vitamin D and hair loss were searched in PubMed and Google Scholar databases to increase comprehensiveness and transparency of reporting. References within the included articles were also reviewed, and the corresponding abstracts and full articles were accessed if found relevant. Some articles were accessed by direct correspondence with the authors. 

Studies that were related to vitamin D and androgenic alopecia were included in this paper. Inclusion criteria were all the types of studies performed within the last 20 years, which included healthy patients with AGA, who were below 50 years of age, were included. There was no restriction on the area of publication, race, or language of publication. It was confirmed that these patients were not suffering from any of the conditions described below.

Eligibility for inclusion in this review was based on AGA specifically. We excluded studies with no relevant data, patients above 50 years of age, patients undergoing chemotherapy, or patients with other underlying autoimmune diseases or gastrointestinal disorders, vitiligo, parathyroid disorders, trichotillomania, and other hair or scalp disorders. 

Results

Online databases (PubMed, Google Scholar, Medline) were searched with the keywords ‘androgenetic alopecia’ and ‘serum vitamin D’. Out of 314 search results that showed up, 12 articles were taken into the study after the application of inclusion and exclusion criteria. 

Discussion

Fawzi et al. evaluated VDRs in patients of alopecia areata and androgenetic alopecia where blood and lesional scalp biopsies were taken from them. It was found that VDR levels in the scalp and blood of patients with AGA and AA were significantly lower [20]. A study conducted by Sanke et al. suggests that vitamin D may play a role in the premature onset of androgenetic alopecia. Hence, vitamin D levels should be assessed in AGA patients [21]. In a cross-sectional study by Kondrakhina et al., the plasma content of trace elements and vitamins was evaluated. It was found that patients with high circulating levels of androgens (dihydrotestosterone) presenting with AGA were deficient in all trace elements and vitamins, including vitamin D [22]. Conic et al. also found lower serum vitamin D levels in patients with AGA compared to controls. [23]. A case-control study by Jun Zhao et al. aimed to evaluate serum vitamin D levels in Chinese patients with different types of alopecia, including AGA. The correlation between low serum vitamin D level and male AGA was found to be statistically significant (P=0.0005) [24]. In a Turkish study conducted by Gulbahar Sarac et al., a correlation was again found between AGA, telogen effluvium, and low serum vitamin D [25]. Danane et al. evaluated the same factors in a tertiary care Indian hospital, and around 82% of AGA patients were found to be vitamin D deficient [26]. In another study including 60 subjects (30 patients of AGA and 30 age-matched healthy controls), the mean serum vitamin D of patients with AGA was 37.1ng/ml compared to controls having 44.2mg/ml level. This is statistically significant (p=0.02) [27]. 

After establishing this correlation, we researched vitamin D as a novel treatment for AGA. It was found that a vitamin D analog (calcipotriol) is effective to cause hair growth in nude mice [28]. A novel oral supplement containing vitamin D given to patients with AGA appeared to increase hair mass index, so it can be considered an adjunct to the treatment of AGA [29]. In a study conducted on mice, VDR expression was studied immunohistochemically. VDR was found to be expressed in the outer root sheath keratinocytes and dermal papilla cells [30].

Vitamin D in Post-Natal Hair Cycling

It has been established that VDR is present in the hair follicles [19]. VDR is responsible for Hr (hairless) gene regulation expression. Destruction of hair follicles during the first catagen is due to increased expression of the Hr gene in VDR null mice [31]. Although the role of VDR is not significant in the development of the hair follicle, it is necessary for the subsequent recycling of the hair follicle [18]. It was demonstrated that in VDR null mice, the hair follicles in catagen become dystrophic, and the dermal papilla separates from the rest of the hair follicle as catagen progresses; consequently, anagen is not reinitiated [31].

Therefore, it can be said that VDR is necessary for anagen initiation. It was also demonstrated that a mutation in the VDR in mice, and mutation in the human homolog, both resulted in alopecia universalis [32]. Since the role of VDR has been established, we can also assume the role of vitamin D in the hair cycle. The same was found that when calbindin D9-k female dogs were given high calcium, high vitamin D diet, their pups did not develop alopecia [33]. Table 1 shows the studies included in our review.

**Table 1:** the studies showing the relationship between vitamin D and hair loss. AGA: androgenetic alopecia; AA: alopecia areata

Publication	Type of study	Year of publication	Number of patients (N=?)	Probability (p=?)	Research question	Conclusion
Fawzi et al. [20]	Case control study	2016	N=20	P=0.000	To assess VDRs in the skin and blood of AA and AGA patients.	Serum and tissue VDR were lower in patients with AGA than controls.
Sanke et al. [21]	Case control study	2020	N=50	P<0.001	To find a correlation between serum vitamin D levels and the severity of AGA.	Significant correlation between vitamin D deficiency and AGA.
Kondrakhina et al. [22]	Cross sectional study	2020	N=50	P<0.001	To estimate plasma element content and vitamin status in patients with AGA.	Multiple micronutrient deficiencies are present in patients with AGA.
Conic et al. [23]	Author manuscript	2019	N=73	P=0.051	To evaluate vitamin D status in scarring and non-scarring alopecia.	Serum vitamin D was low in AGA patients.
Jun Zhao et al. [24]	Case control study	2020	N=777	P=0.0005	To evaluate the serum vitamin D status in Chinese patients with AGA.	Association between serum 25(OH)D levels and AGA in a Chinese population.
Sarac et al. [25]	Case control study	2018	N=58	P=0.01	To determine the role of vitamin D in hair loss	Patients with AGA were vitamin D deficient.
Danane et al. [26]	Longitudinal Follow up	2021	N=50	P value not defined.	To measure the vitamin D levels in men with premature AGA and to demonstrate its relationship with the severity of the disease.	Significant correlation between vitamin D deficiency and the severity of AGA.
El-Tahlawy et al. [27]	Case control study	2021	N=30	P=0.02	To measure serum vitamin D and serum ferritin levels in patients with male pattern hair loss.	Vitamin D was statistically significantly lower in patients with AGA.
Narang et al. [28]	Prospective study	2017	N=22	P<0.009	To study the efficacy of calcipotriol lotion 0.005% in AA and correlate its outcome with serum vitamin D levels.	Hair growth was observed to be better in patients with low serum vitamin D levels.
Nichols et al. [29]	Case series	2017	N=10	For THC: P=0.014 For HMI: P=0.003	To evaluate the effectiveness of a novel oral supplement containing vitamin D.	This novel supplement may be a useful adjunct in the treatment of AGA.
Bilke et al. [31]	Animal study	2006	N/A	N/A	To evaluate if new hair cycle has started in the VDR null mice.	Lack of VDR causes disruption of hair follicle structure during the first catagen resulting in failure of subsequent hair follicle cycling.
Mady et al. [33]	Animal study	2016	N/A	N/A	The role for 1,25-dihydroxyvitamin D_3_ and/or calcium in hair follicle cycling by evaluation of alopecia in calbindin-D_9k_knockout pups.	In calbindin-D_9k_knockout pups, a maternal vitamin D-deficient/low-calcium diet leads to transient non-cicatricial alopecia.

Male and Female Pattern Androgenetic Alopecia

AGA is of two types, namely the male pattern and the female pattern. Both have a different characteristic pattern of hair loss. The female pattern differs from the male pattern, as females experience thinning of hair, which does not start at the hairline. Female pattern hair loss (FPHL), like male pattern hair loss (MPHL), is often associated with hyperandrogenic conditions. MPHL has often been termed the PCOS (polycystic ovary syndrome) of men [34]. Similarly, patients presenting with FPHL are most often diagnosed with PCOS. In a study conducted in England, 67% of the FPHL patients had PCOS, while 27% in the control group had the condition [35]. 

Having established the similarities between MPHL and FPHL, we searched the prevalence of low serum vitamin D levels in female patients of AGA. In one case-control study, it was found that FPHL patients had much lower serum vitamin D3 levels than controls (P=0.004) [36]. In another study by Rasheed et al., serum vitamin D2 levels were also found to be lower in patients of FPHL than controls [37].

While we investigated the low serum vitamin D levels in MPHL, we discovered that it might also be an important diagnostic parameter in FPHL. 

Deficiency of Other Vitamins and Trace Minerals

One of our studies found that along with vitamin D, the patients with AGA also had lower levels of zinc, copper, magnesium, selenium, and vitamin B12 [22]. It is a well-known fact that a healthy, well-balanced diet is essential for healthy hair and to delay the signs of ageing. Vitamin B complex, vitamins E, A, C; iron, zinc, magnesium, are all important for delaying the onset and severity of androgenetic alopecia. Jin, Zhu, and Wu compared zinc, copper, iron, and manganese contents in the hair of patients with male pattern alopecia and healthy men confirming low levels of nutrients in patterned hair loss [38]. In fact, supplemental treatments with zinc, marine extract, melatonin, caffeine, and biotin have been shown to be effective against AGA [39]. Hence, it can be said that patients suffering from vitamin-D-related hair loss may also have other nutritional deficiencies. 

The role of vitamin D has been well established in AA, which is a hair follicle-specific autoimmune disease. Systematic review and analysis by Gerkowickz et al. showed the correlation between vitamin D deficiency and AA. It analyzed a total of 14 studies. Vitamin D deficiency was found to be prevalent among all the patients suffering from AA. It also proved to be an important diagnostic parameter as this deficiency was a common finding among patients suffering from AA, FPHL, and telogen effluvium [40]. 

Increased Prevalence of Vitamin D Deficiency

Vitamin D deficiency and insufficiency is a global health issue that afflicts more than one billion children and adults worldwide [41]. It has become common due to several factors such as increased use and awareness of sunscreen, increased time spent indoors, ageing (a decreased precursor in keratinocytes), decreased intake of milk, obesity, and use of certain medicines [42]. Vitamin D deficiency adversely affects bone health and calcium homeostasis. This leads to diminished bone mineralization and an increased risk of fractures. Vitamin D is also linked to other diseases, including cancer, autoimmune diseases, myocardial diseases, and muscle weakness [43]. It can be corrected by supplementation. The Institute of Medicine recommends 600 IU of vitamin D per day for people aged 1 to 70 [44]. Since it is difficult to obtain this only through diet and exposure to sunlight, it is recommended to take vitamin D supplements to maintain adequate plasma concentration [45]. 

*Efficacy of Vitamin D to Treat Androgenetic Alopecia:* 

In a study conducted by Nichols et al. on patients with AA who were given a supplement containing cholecalciferol, a positive correlation was found between improvement in terminal hair count and hair mass index [29]. There have been a few studies exploring the use of oral and topical vitamin D as a possible treatment for different types of alopecia, including AA. In a study conducted on nude mice, it was found by histological examination that supplementation with vitamin D3 analogues stimulated the development of hair follicles [46]. However, oral vitamin D led to hypercalcemia in human subjects [47]. Therefore, we explored studies that tested topical vitamin D3 analogues on human subjects. In a study conducted in 2015, 48 patients were given calcipotriol topically for 12 weeks. In the end, there was a positive response in 69.2% of patients (P=0.001) [48]. Furthermore, in a pilot study conducted by Narang et al., it was found that patients treated with topical calcipotriol responded well, especially those with a vitamin D deficiency, as measured by their serum vitamin D levels before and after the treatment [28]. The efficacy and safety of vitamin D and its analogues as a possible treatment for AA are yet to be determined. Most authors agree that supplementation with topical and oral vitamin D significantly improves the status of AGA and telogen effluvium [49].

Limitations

First, there are a limited number of research articles exploring the exact effects of vitamin D on AGA, although extensive research has been made about the correlation between serum vitamin D and other types of alopecia, such as AA and FPHL. 

Second, this is a traditional review of previously published work; hence, there is an increased risk of bias. Since we found a limited number of articles exploring vitamin D supplementation in human subjects with AGA, we had to include animal studies in our review. 

## Conclusions

From our review of available literature, we can conclude that in healthy male subjects presenting with AGA and who are not suffering from any systemic illness leading to AGA, serum vitamin D levels are an important parameter for diagnosis and possible treatment. Based on this review, we suggest that using vitamin D3 analogues topically on human subjects could be a possible treatment for AGA. However, additional investigation is required about the use of vitamin D alternatives orally for AGA. It may be considered that serum vitamin D could be a parameter for diagnosing the onset and severity of AGA.
